# Computed tomography-guided vertebroplasty using a stereotactic guidance system (stereo-guide)

**DOI:** 10.4103/2152-7806.63912

**Published:** 2010-05-31

**Authors:** Arun Angelo Patil

**Affiliations:** Professor of Neurosurgery, University of Nebraska Medical Center, Omaha, NE, USA

**Keywords:** Computed tomography guidance, stereotactic, vertebral fracture, vertebroplasty

## Abstract

**Background:**

In order to make it easy to perform computed tomography (CT)-guided vertebroplasty a stereotactic guidance system called the "stereo-guide" was designed. A method to perform CT-guided vertebroplasty using this system is described.

**Methods:**

The device is a rectangular flat plastic block. One of the flat surfaces of the block has deeply grooved protractor markings at 5-degree intervals; ranging from 0 to 30 degrees. The procedure is performed on the CT table. Based on distances and angle measurements obtained from CT images the device is placed on an appropriate location on the back of the patient and the needle is advanced to the target through the pedicle guided by the grooves on the device. Ten procedures were performed in nine patients with lumbar and thoracic pathology.

**Results:**

The system was easy to use and proved to be accurate. No complication resulted from the procedure.

**Conclusion:**

The stereo-guide proved to be simple and easy to use. Intraoperative scans helped to plan the trajectory and follow the injection of the cement.

## INTRODUCTION

Vertebroplasty is a common procedure for compression fractures of vertebral bodies associated with pain.[4] The procedure can be performed under fluoroscopic control or with intraoperative computed tomography (CT) scans.[1,2,3,5,6] Both these procedures are done using free hand technique. In order to make it easy to perform CT-guided vertebroplasty, a stereotactic guidance system called the “stereo-guide” was designed. This paper describes the device, the method to use it and initial experience with it.

## MATERIAL AND METHODS

### Description of the device

**Figure 1:**
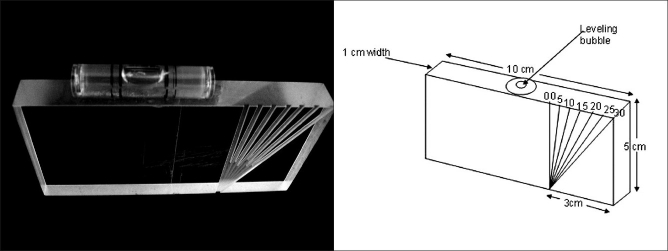
a: Photograph of the system b: Diagram of the system showing the dimensions

The device [Figure 1] (manufactured in our machine shop) is a rectangular flat plastic block measuring 10 cm in width, 5 cm in height and 1 cm in thickness. One of the flat surfaces of the block has deeply grooved protractor markings at 5-degree intervals; ranging from 0 to 30 degrees. The grooves are deep and wide enough to snuggly accommodate an 11-gauge Jamshidi bone biopsy needle. A leveling bubble is located on the top surface of the device.

## METHOD

**Figure 2:**
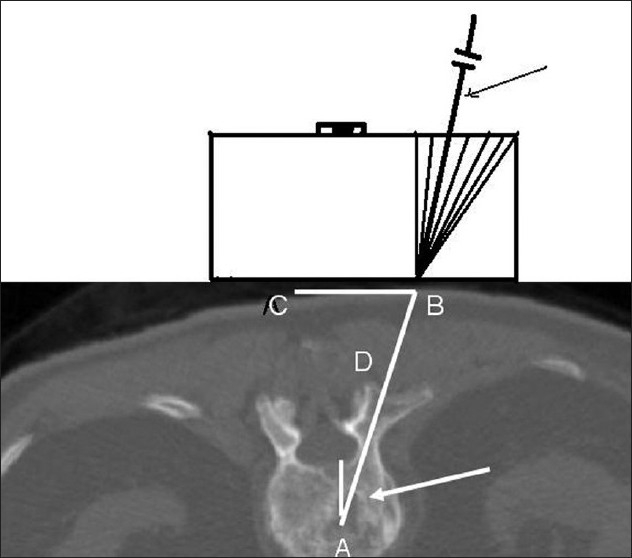
A sketch of the stereotactic guide positioned on CT axial image obtained during needle insertion. A-the target point; CB-distance from the midline to entry point of the needle on the skin surface; BD-distance from the skin surface to the top of the pedicle; BA- the distance of the trajectory from the skin surface to the target; and angle at A-angle of the trajectory with the vertical; white arrow-trajectory of the needle; black arrow shows the needle positioned in the appropriate groove in the stereo-guide. Note that the stereo-guide is held perfectly horizontal in the coronal plane

**Figure 3:**
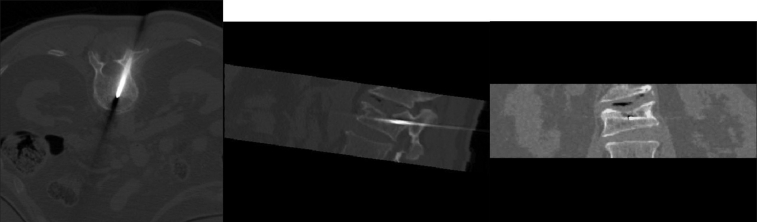
a: The needle tip at the target in axial view b: Needle tip at the target in saggittal view c: Needle tip at the target in coronal view

**Figure 4:**
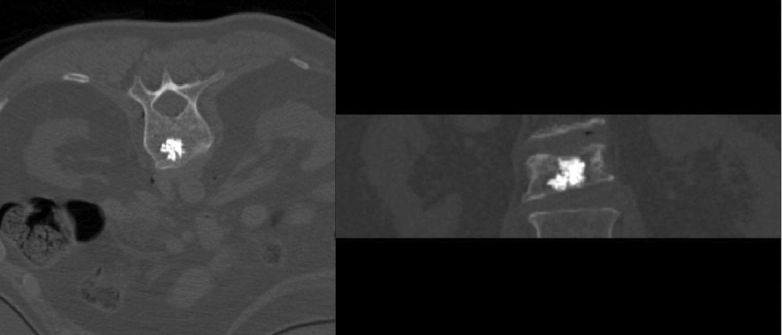
a: xial view after cement injection b: Coronal view after cement injection

The patient was placed prone on the CT table under general anesthesia. The table height was adjusted so as to place the patient approximately at the isocenter of the gantry. This was necessary in order to obtain scans with a tilt in the gantry. A lateral scout image was obtained. The angle of the pedicles with the vertical was measured. The gantry was tilted to this angle. Serial CT scans were obtained in the area of interest with a scan thickness and interval of 2 millimeters. The number of scans through the pedicle was counted and the middle one was chosen for distance measurements [Figure 2]. Entry to the target was through only one of the pedicles. There was no preference to the side of entry. Using the distance measurement mode of the CT computer, the length of line from the target through the middle of the pedicle to the skin surface was measured. The point made by this line on the skin surface was chosen as the entry point for the needle. The angle of this line with the vertical line and the distance of this line from the skin surface to the top of the pedicle were measured. Then the distance from the entry point to the midline was measured. A line was drawn on the patient's back to mark the midline. The CT table was then moved to bring the plane of the target in the plane of the horizontal laser light of the scanner. The needle entry point was marked with a small stab wound on the line created by the laser light on the patient's back at distance of CB [in Figure 2]. The device was then held in position, perfectly horizontal in the coronal plane (using the leveling bubble) with the lower end of the grooved surface along this line and the convergent point of the grooves at the stab wound [Figure 2]. The stereo-guide was then tilted in the sagittal plane parallel to the gantry. The distances AD and AB in Figure 2 were measured from the tip of the needle and marked. An 11-gauge Jamshidi bone biopsy needle was placed in the appropriate groove (at an angle approximately equal to the angle at point A in Figure 2) on the stereo-guide and advanced to the target. Bony resistance was encountered when the deeper marking on the needle reached the skin surface. If this did not occur, the trajectory of the needle was slightly altered to find the bony surface. A mallet was used to insert the needle in the bone. Intraoperative scans were obtained to follow the progression of needle insertion. If needed, necessary corrections were made in the trajectory of the needle. Once the target was reached [Figure 3] cement was injected. During injection, a series of scans were obtained [Figure 4] to make sure the cement was heading in the right direction.

## RESULTS

Ten procedures were performed on nine patients without any complication and good relief of pain in all. All procedures were done through one pedicle. The male/female ratio was 3/6 and age range was 50-70 years. The follow-up is between 7-31 months with a median of 26 months. One patient had two procedures at two different times (one at L2 and another at T10). Six procedures were at L1; two at L2; and one each at L3 and T10.

## DISCUSSION

This is a simple device with straightforward methodology. In our small experience it was found to be useful. Since it is handheld it can introduce errors. However, because intraoperative scans are obtained these errors are corrected to get accurate placement of the needle. Furthermore, the procedures were done under general anesthesia to prevent patient movement. The system could be improved by having a rigid system to hold it in place; and by having a rigid probe holder.

Though fluoroscopic images offer a straightforward technique, the distinct advantage of CT images is that they provide images in the axial, coronal and sagittal planes. In addition, we were able to have entry to the target through only one pedicle; because it is easy to plan a trajectory to a target close to the middle of the vertebral body using this device.

There may be concern about how quickly one can obtain scans while following the entry of the cement into the vertebral body. Fortunately, most modern scanners are very fast and have a viewing screen in the scanner room. It is, therefore, possible to obtain a successive series of scans and observe the flow of the cement in the scanner room during injection. In addition, one can also use the CT-fluoroscopic mode.

Most CT scanners can display images in all three planes almost immediately after the scans are obtained. Therefore, display of the needle in all three planes as it is being advanced to the target makes this system particularly useful for patients who have severe compression fractures. Similarly, it also makes it easy to follow the flow of the cement in all three planes, especially when there is concern of flow of cement into the spinal canal in patients who have minimal retropulsion of fractured segment.

In summary, a simple device and methodology for CT-guided vertebroplasty is described. Use of intraoperative CT scans made this procedure accurate. Furthermore, axial CT images enabled us to perform the procedure through a single pedicle.
